# Following the *Bitis* pet trade: a practice-based exploration across Africa and Europe

**DOI:** 10.1007/s42532-026-00246-3

**Published:** 2026-03-12

**Authors:** Janine Brigitta Heim

**Affiliations:** 1 Criminal Law and Criminology, Maastricht University, Maastricht, Netherlands; 2 Global Risk Governance Programme, University of Cape Town, Cape Town, South Africa

**Keywords:** Exotic pet trade, Human-wildlife interactions, Environmental governance, Conservation, Value-making

## Abstract

In this article, I investigate the trade dynamics of *Bitis*, a genus of African vipers, within the exotic pet market, with a particular focus on trade flows between South Africa and Europe. The conservation status of the 18 recognised *Bitis* species ranges from Least Concern to Endangered, with official assessments primarily attributing threats to environmental destruction. Employing Nicolini’s “zooming-in, zooming-out” method, I trace the practice of trading *Bitis* through netnography, in-depth interviews, and field visits to examine the socio-ecological dimensions of this semi-regulated trade. This article presents the findings of this multi-sited research. I outline the mechanics of the trade, which, despite being largely legal, often involves grey and illegal activities due to regulatory ambiguity and inconsistency. The underlying value structures driving the trade include the ornamental appeal of *Bitis*, along with secondary motivations such as its behavioural traits, sentimental value, and perceived rarity. I show how these value-making practices co-produce the socio-ecological conditions under which *Bitis* are collected, bred, and traded, and how these conditions can undermine or enable attempts to regulate the trade.

## Introduction

Wildlife and exotic pet trades have come under intense academic scrutiny primarily within the context of conservation biology (Duffy 2022a, pp. 25–30; Fukushima et al. 2021, pp. 2–4; Rivera et al. 2024; Roe et al. 2020), yet further research is need to foster a broader understanding of the motivations and dynamics that underpin these trades. Digging deeper into this field, this paper focuses on a particular subsection of the exotic pet trade: the trade in *Bitis*, a genus of African vipers. South Africa is home to 12 of the 18 *Bitis* species, with some occupying highly specialised habitats while others occur across the African continent (Barlow et al. 2019, pp. 1235–1237; Gonçalves et al. 2019; Gower et al. 2016; Maritz and Alexander 2012). Their conservation status varies from Least Concern to Endangered (“The IUCN Red List of Threatened Species 2024; Spawls 2014, pp. 2–4). While some countries such as South Africa have strict regulations on the trade in *Bitis*, others, such as Togo, home to three *Bitis* species, have more relaxed rules (D’Cruze et al. 2020, pp. 78–84; Harrington et al. 2021, pp. 8–10; Segniagbeto et al. 2011, pp. 351–352).

In this paper, I aim to unravel the intricate values, motives, and practices that drive the *Bitis* trade. By focusing on a niche trade, I deviate from the dominant body of wildlife trade research, which focuses on high-value wildlife commodities such as trophy, medicinal, and fashion trades (‘T Sas-Rolfes et al. 2019, pp. 217–218; Anagnostou and Doberstein 2022, pp. 1621–1624; Hinsley et al. 2024, p. 5), and broaden the lens to include small-scale, but equally complex and significant, trades in wildlife as exotic pets. Here, I critically examine the value placed on these *Bitis* as pets by various actors in the trade’s structure, including keepers, breeders, amateur herpetologists, and a range of service providers. I found value to be socially constructed and continuously negotiated among both participants in the trade and other stakeholders, such as conservationists and regulators. Rather than adopting the traditional narrative that portrays wildlife traders and collectors as threats to conservation, the paper examines them as key contributors to the creation of value within the *Bitis* community.

My overarching research questions are: (1) How does the Bitis trade operate in practice, both materially and within regulatory frameworks? (2) what exactly is being valued, by whom, and through which practices? and (3) how might the results help improve governance while also reflecting the socio-ecological realities of the trade? To answer these questions, I observed trade across virtual and physical spaces, using the zooming-in, zooming-out approach (Nicolini 2009). In the following article, I examine the results of these observations through using thematic analysis. I begin by exploring the mechanics of the *Bitis* trade and subsequently extend my analysis to consider the underlying value constructions that shape and sustain it. Examining both of these aspects is essential given the diverse ways in which humans engage with wildlife, encompassing the desire to collect, breed and possess species like *Bitis*. This allows the examination of the “webs of significance” (Geertz 1973) that shape value in the *Bitis* trade.

This approach also underscores the intricate web of interactions, practices, and knowledge through which wildlife users and conservationists move beyond the binaries of villain or hero in species conservation (Duffy 2022b, p. 15). I aim to contribute information on the trade of a neglected genus of animals, one of many such genera, as pointed out by Hinsley et al. (2024, pp. 4–6), to complement ecological data aimed at preserving wildlife and managing its trade. By outlining how both trade structures and value systems shape socio-ecological realities around *Bitis*, this study provides insights that can help regulators, conservation practitioners, and trade participants navigate the areas where ecological concerns, market dynamics, and legal frameworks intersect. While centred on *Bitis*, it demonstrates an approach to reconstructing niche wildlife trades that operate through small communities, inconsistent legal frameworks, and complex, value-driven exchanges. In this sense, the results are intended to support more context-sensitive debates on oversight, monitoring, and species management.

## Research methods

Utilising Nicolini’s (2009) zooming-in, zooming-out approach, I explored the complex, interconnected network of practices in the *Bitis* trade. This approach draws on practice theory and related traditions and traces the interconnectedness between various practices by oscillating between the execution of specific practices in distinct locations (zooming-in) and their associations and effects when connected in different ways (zooming-out) (Nicolini 2009, p. 1408). With this method, I examined *Bitis* practices within their immediate context (zooming-in) and a broader network and influence realm (zooming-out). I adapted the zooming-in, zooming-out approach to focus on three aspects: scrutinising the connections between virtual and physical spaces, exploring the trade from an integrated network perspective without segregating legal from illegal aspects and applying various theoretical perspectives to comprehend *Bitis* trading practices.

I collected data from August 2021 until December 2023. This includes netnography-based online observations on social media, participation in international exotic pet exhibitions and interviews with stakeholders in the *Bitis* trade. I obtained ethical approval for this project from Maastricht University’s Ethics Review Committee Inner City faculties (ERCIC), the European Research Council (ERC) and the Faculty of Law Research Ethics Committee (REC) at the University of Cape Town. Throughout my online and physical fieldwork, I disclosed my identity as an academic researcher to all participants and procured informed consent for all formal interviews. Regarding any closed social media groups, I announced my researcher identity to group moderators and declared it on my profile, with details about my research included in posts and associated links.

For this study, I employed numerous sources to bridge data gaps, counter biases, and consolidate the extensive knowledge I gathered through the use of netnography, semi-structured interviews and field visits. My research began with observational studies of the *Bitis* community online, which involved participating in various specialised Facebook groups and following key community figures across multiple social media platforms, resulting in over 500 observations. I conducted these observations across different platforms: Facebook, Instagram, TikTok, and several forums and blogs, such as terraristik.com, SA reptiles, Venomland, and speciestrade.com, and blogs of specific individuals. Following my online observations, I conducted 19 formal, semi-structured interviews with key figures involved in the *Bitis* trade, ranging from keepers, breeders and veterinarians to biologists, conservationists and other service providers (such as couriers, terrarium suppliers and insurance providers); many of whom held multiple roles simultaneously. I identified interviewees through my online observations, during which prominent keepers, breeders, hobby herpetologists, and others involved with *Bitis*, whether professionally or as amateurs, quickly became visible. Several potential participants declined, often due to the perceived political sensitivity of the subject, while others had scheduling conflicts. Those who agreed to take part sometimes recommended additional individuals, which resulted in a small amount of snowball sampling. The interviews were guided by a checklist organised around three main themes: general breeding practices, motivations, and the wider *Bitis* community (see Semi-Structured Interview Checklist in Appendix). However, the interview format remained flexible and participant-driven. This approach allowed interviewees to share their experiences in their own words, while also helping to minimise potential bias introduced from the interviewer. Nine interviewees were based in Europe, mainly in the DACH (Germany, Austria, Switzerland) region and Northwestern Europe. The remaining ten were linked to southern Africa, including South Africa and Lesotho. Several participants divided their time between more than one country. I supplemented these formal interactions with informal dialogues and online discussions with a wider group of individuals, including additional breeders, veterinarians, sanctuary workers, and casual expo attendees, among others. These conversations provided significant context for my online observations and supplied diverse perspectives, insights, and assessments related to these as well as my in-person observations. This online fieldwork also enabled me to identify reptile trade fairs and localised regional meetings in Europe. These included Terraristika in Hamm, Germany, one of the largest international reptile trade fairs, Terraria and Snakeday in Houten, Netherlands, both large European reptile expos and Reptilienbörse Dreiländereck in Tettnang, Germany, a smaller, regional reptile trade fair. Supplementary research was conducted at the archival repository of the Royal Zoological Society of London, which contains unique historical data on the *Bitis* trade. Four field visits were made to South African zoos: Reptile Garden, Exotic Animal World, Cape Point Ostrich Farm & Snake Park and Reptile World. Additional data sources include grey literature; niche publications such as exotic pet-keeper magazines; Convention on International Trade in Endangered Species of Wild Fauna and Flora (CITES) records^1^; Wildlife Trade Portal data collected by TRAFFIC, International Union for Conservation of Nature (IUCN); South African National Biodiversity Institute (SANBI) assessments; and archival records kept by the London Zoological Society. The use of diverse data sources not only addresses gaps in previous research but also enables a more comprehensive understanding of traded species and their supply chains (El Bizri et al. 2024, pp. 3–5; Hatten et al. 2024, pp. 3–7; Hu et al. 2024, pp. 5–10). The research questions emerged inductively during these stages. Initial online observations highlighted recurring points of tension and ambiguity in terms of how value was discussed, prompting the refinement of questions to focus on value-making processes and trade practices. Interviews and field visits further shaped the questions by revealing moral, regulatory, and experiential dimensions absent from the existing wildlife-trade literature.

Data were systematically coded and organised using MAXQDA software. To ensure alignment between digital and physical materials, traditional highlighting and annotation techniques were applied to artefacts in parallel with digital coding. After I had familiarised myself with the data and completed iterative coding rounds, the codes were grouped according to the practical elements of *Bitis* trading and the broader webs of value that shape this context. The analytical approach followed grounded, inductive reasoning: recurring patterns of meaning were distilled into themes corresponding to practical, geographic, and regulatory dimensions, the mechanics of the trade, and matters of motivation, moral perception, and desire, which inform the underlying values driving the trade. Given the complexity of the subject matter, these thematic domains often overlapped. This analysis utilised both semantic and latent coding strategies.

**Fig. 1:**
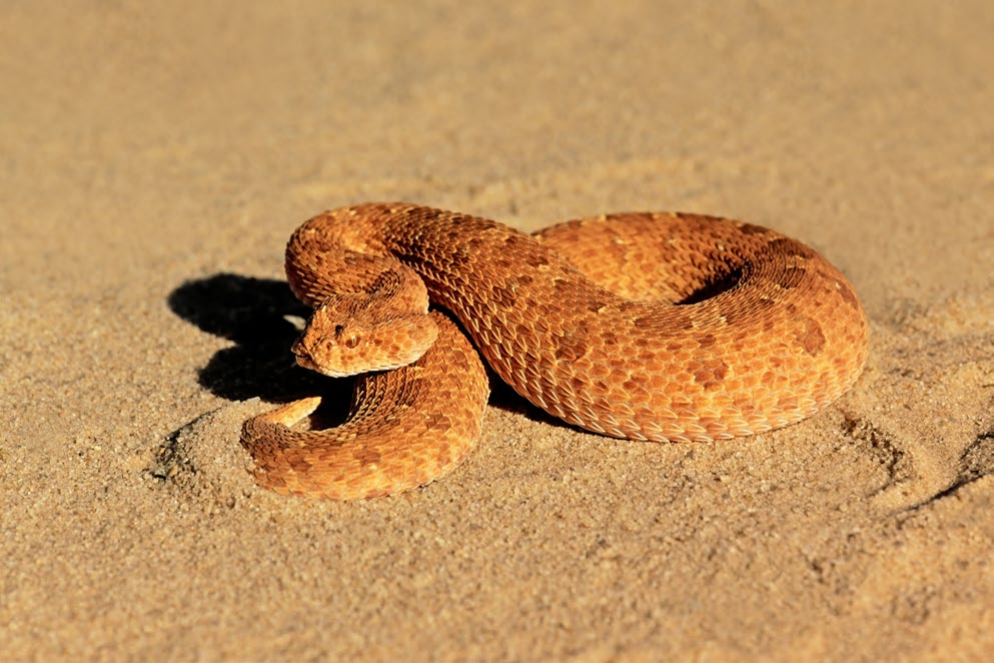
*Bitis caudalis* from the Kalahari Desert

**Fig. 2:**
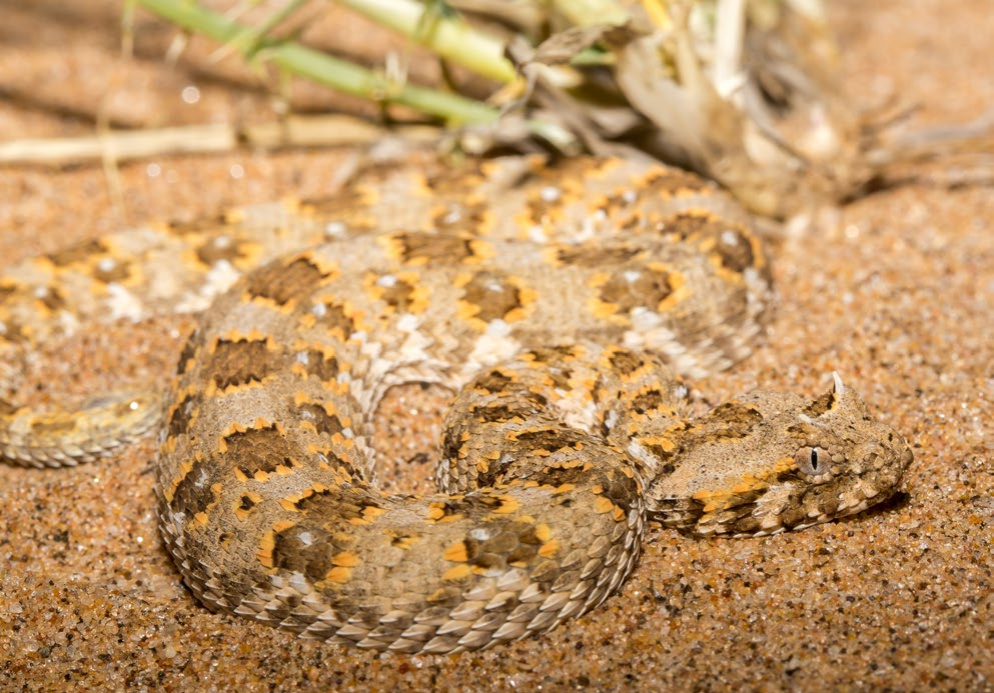
*Bitis caudalis* from the Namib Desert

This process produced a cohesive analytical framework for interpreting both material artefacts and discursive data. I generated themes inductively from repeated patterns identified in my collected observations and refined them deductively in dialogue with theoretical perspectives on il/legality, morality, and values. All the coding resulted in the following broad patterns of meaning, each comprising a wide range of detailed subcodes: *Breeding*,* Keeping and Related Trading Practices*, capturing diverse breeding practices and standards, trade dynamics, and reflections on animal welfare; *Security and Risk*, encompassing multiple forms of perceived risk, including threats to both snakes and humans; *Value Constructions*, addressing varied motivations for keeping *Bitis*, shifting perceptions of worth, and pricing references, including aesthetic preferences, trends, rarity, locality purity, and morphological traits; *Il/legality and Morality*, covering nuanced discussions of wild collection, legality and rule-following, and the moral reasoning behind boundary-crossing practices; and *Geographic and Species-Specific Classifications*, including extensive locality references and a detailed taxonomy of *Bitis* species. I explore these themes, their components, and their relationships in Sect.  3 (Observations on the Mechanics of the Trade) and 4 (Observations on Perceptions of Value in the Trade). I visualised the geographical data using Excel and QGIS (see Figs. 1 and 2).

## Observations on the mechanics of the trade

The trade in *Bitis* is a niche market. Because of its small size and complicated legal landscape, it has interweaving legal, grey, and illegal aspects; in this way, it is similar to many other international trades, such as those in gold mining (High 2012, p. 250), olive oil, and meat (Gregson and Crang 2017, pp. 213–215). I found the *Bitis* trade to be sustained by a tightly knit infrastructure of specialised expertise, informal networks, and uneven regulatory regimes, through which animals move across legal, grey, and illegal spheres. These mechanics reflect several intersecting categories that emerged during coding; these were practical, logistical and regulatory elements that appeared as recurrent patterns across the dataset. Because similar mechanics may also occur across other niche exotic pet markets, I suggest that aspects of this analysis may be informative for researchers exploring value systems in other niche trades guided by similar laws, regulations, and geographical factors. Reconstructing these mechanics has practical implications, as it highlights the specific points at which regulatory provisions are either effective, inconsistently applied, or strategically circumvented. Furthermore, I see the mechanics I observed as the skeleton of the trade: it is fundamental to how each part connects and moves. Understanding this part of the *Bitis* trade anatomy is essential to situating the motivations and interactions that shape this market and provides the foundation for the value‑centred analysis that follows.

### Centres of breeding expertise

*Bitis* breeding expertise is principally found in Europe, particularly the DACH area and South Africa, with fewer than ten active and dedicated *Bitis* breeders worldwide. In Europe, captive breeding operates within a fragmented regulatory landscape: international trade in most *Bitis* species is not subject to CITES controls, and there is no EU-wide equivalent to the U.S. Lacey Act, which restricts trade in illegally sourced wildlife. Instead, rules on keeping and breeding venomous snakes vary considerably across and within EU states, with federal systems such as Germany’s producing patchworks of regulation and enforcement, in contrast to national permit systems such as Switzerland’s (Eurogroup for Animals, 2020, pp. 20–36; Gebhardt-Brinkhaus 2023; Schweizerische Bundesrat, 2008).

My observations include photos and videos shared by breeders on social media which document the successful breeding of various *Bitis* species by private individuals. These species included *Bitis armata*, *Bitis arietans*,* Bitis atropos*,* Bitis caudalis*,* Bitis cornuta*,* Bitis nasicornis*,* Bitis rhinoceros*,* Bitis rubida*,* Bitis schneideri*,* Bitis worthingtoni* and *Bitis xeropaga*. Most recently, Kane et al. (2022) documented the successful breeding of *Bitis parviocula* at London Zoo. Less detailed reports of successful private captive breeding (Maritz et al. 2013). *Bitis* are primarily sold through social media, personal networks and exotic pet shows. The DACH region often exports internationally, including to the United States, Eastern Europe, and South Africa.

### Practices of collecting *Bitis* from the wild

The majority of interviewees believed that the initial collection of *Bitis* from the wild, from which the first captive-bred specimen might have descended, took place in South Africa, Namibia, Ethiopia and Kenya from the 1960s to the 1980s. The general belief among interviewees was that large-scale poaching now belongs to the past, attributing the decline of such activities to the advent of successful captive breeding. However, numerous interviewees candidly recognised that poaching, although less rampant, has not been fully eliminated.

Wyatt distinguishes between “subsistence harvesters,” “opportunistic harvesters,” and “specialist harvesters [of wildlife]” (2021, pp. 127–129). Most individuals involved in capturing *Bitis* snakes from the wild typically possess a high degree of specialisation in this genus of African reptiles and possibly others, or at a minimum, in reptiles endemic to South Africa. The need for such specialisation is crucial, as *Bitis* pose logistical challenges due to the inherent risk of venomous bites. According to my interview and observational data, this residual small-scale poaching appears to primarily focus on small-bodied *Bitis* in Namibia and South Africa as well as the Ethiopian *Bitis parviocula*. *Bitis arietans*, the most common *Bitis* in the wild (Barlow et al. 2019, p. 1236), are seldom targeted, with the possible exception of those hailing from Kenya due to their impressive orange and yellow colouring.

Based on my online observations, it appears that interviews and field visits to sanctuaries, several factors appear to limit current wild collection, including the time, resources, and physical effort required to access desert- or mountain-dwelling *Bitis*. An often-practised method is road cruising for snakes, which consists of looking out for snakes while driving (Blais et al. 2024, pp. 3–4); some individuals not just observe but also collect *Bitis*. Temporary practices of collecting *Bitis* from the wild are contentious within the community. Collecting snakes from oncoming traffic is debated, with some viewing it as rescue due to the risk of snakes being killed by vehicles, while others argue that even dead snakes have ecological value and should not be removed. Other forms of wild collection include planned, targeted trips to specific regions with the explicit intent to collect *Bitis*, which interviewees reported having witnessed or known about through the community. Another form of wild collection involves snakes that appear on private property in some South African cities, such as Gqeberha (often referred to as Port Elizabeth in the *Bitis* trade), and are collected.

A bone of contention that emerged during multiple interviews was the possibility of gravid *Bitis* being collected from the wild, a concern for conservationists. While there was agreement that males are more commonly caught due to increased movement during mating season, opinions differed on gravid females: some South African interviewees suggested they are easier to find because they bask more, while others believed they remain more concealed. At least one documented case of a gravid *Bitis xeropaga* collected from the wild exists (Fleck 1999, p. 6). In South Africa, both resident South Africans of European descent and visiting Europeans were reported to collect *Bitis*, while no accounts describe local South African communities doing so; however, an older account exists of an individual conscripting locals to harvest *Bitis parviocula* in Ethiopia (Smith, 2011, pp. 262–268).

### Regulation, permit systems, and loopholes in source countries

**Fig. 3:**
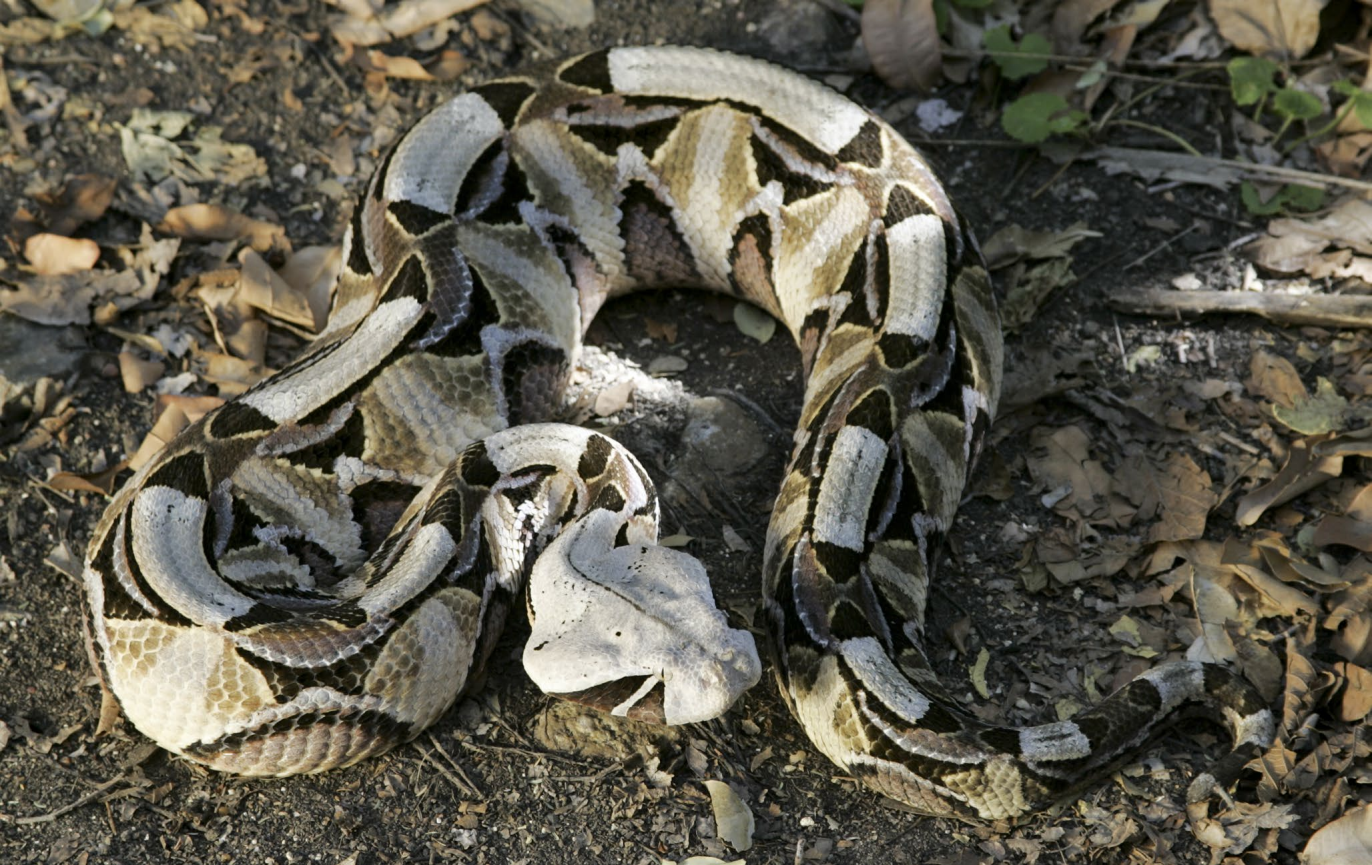
*Bitis gabonica*, similar to *Bitis rhinoceros* in appearance, is the longest and heaviest *Bitis*, with the longest fangs of any venomous snake in the world (Warner 2009, pp. 11–13). As a result of these traits, it is a common feature in venomous snake collections and zoos

The South African provincial permitting system significantly shapes *Bitis* collection and export practices. This regulatory system has had a similar effect on other wildlife trades in that country. Following apartheid, South Africa reformed environmental laws to align with international conservation standards, particularly CITES, which the apartheid regime had only partially observed (Hübschle 2017, pp. 188–191). The democratic government, established in 1994, enacted the National Environmental Management Biodiversity Act (NEMBA) in 2004, followed by the Threatened or Protected Species (TOPS) regulations in 2008, to comply with CITES guidelines (Hübschle 2017, pp. 188–191). Within this regulatory context, the phenomenon of “province hopping” has been noted by Hübschle (2017, pp. 188–191) as a critical challenge, particularly in the regulation of rhino hunting. Province hopping refers to the practice of collectors moving across provincial jurisdictions to circumvent hunting quotas. Despite efforts to centralise oversight to mitigate this issue, disparities persist across provincial enforcement and are exacerbated by logistical and bureaucratic constraints, thereby complicating the effective implementation of the TOPS regulations. I have observed similar behaviours to circumvent some provincial regulations in the *Bitis* trade. The geographical distribution of small-bodied adders spans a variety of habitats in the north-west, west and south-west, with the large-bodied *Bitis arietans* present everywhere and *Bitis gabonica* (Fig. 3)extending into north-eastern South Africa (Barlow 2012, pp. 118–123; Barlow et al. 2019, pp. 1235–1237; Gower et al. 2016, p. 54; Warner 2009, pp. 11–13). Generally, South Africa restricts *Bitis* exports, (Wyk 2023), however, regulations vary from province to province as KwaZulu-Natal can issue permits for the export of Indigenous species (*Government Gazette Staatskoerant* 2023; Marais 2023). *Bitis* species, not native to South Africa, are classified as exotic pets and also require permits for ownership. For endangered *Bitis* species, further legal provisions under the Threatened or Protected Species (TOPS) permits are mandatory. I was told that, for example, once collected in the Western Cape, poached *Bitis* are transported to KwaZulu-Natal, where they can be legally kept and are sometimes mislabelled for export. Given the morphological similarities between small-bodied adders, mislabelling is an effective means of exporting endangered *Bitis* species. Another potential issue with the provincial regulation system that was pointed out by other interviewees was the abuse of permits and the laundering of wild-collected specimens. Distinguishing between captive-bred and wild-caught specimens can be challenging; however, indicators like scars, parasites, and certain behaviours, such as an aversion to dead food, strongly suggest a life spent in the wild.

## Observations on perceptions of value in the trade

These mechanics of the trade both shape and are shaped by the perceived value of *Bitis*. This section elaborates on my findings on how different actors actively construct values around *Bitis*, continuously negotiating value with one another and with regulators, tracing the logics that ultimately underpin trade decisions and broader market dynamics. If the mechanics of the trade are its skeleton, then the values I observed are the muscles that animate it, and are therefore equally important to understanding trade practices. In this section, I examine the complex patterns I observed in my data and interpret them as the values that drive trade, as well as analysing the inherently social construction of these values. This topic weaves together semantic and latent observations from my data, with many strands contributing to the intricate web of values I identified. My central finding is that value in the *Bitis* trade is not a fixed attribute made tangible by prices, but a continuously negotiated and socially produced construct in which monetary worth, locality, aesthetics, behaviour, perceived legitimacy, risk, and symbolic meanings intersect to shape trade practices.

### Monetary value and market volatility

The most tangible component of this complex value structure is the reported selling price of *Bitis*, although the accuracy of the reported prices cannot be substantiated. When discussing prices, I retained the original currencies for clarity: euros for sales in the European Union (EUR) and US dollars for sales in the United States (USD). Breeders reported some price flexibility, signifying a certain degree of market volatility. While rarely displayed, some of the prices I was able to observe for *Bitis* species varied greatly: Table 1

**Table 1:** Observed price ranges of *Bitis* Species, including specific Phenotypes/Localities

Bitis species and phenotypes/localities	Price range (currency as observed)
Bitis arietans	100–300 EUR
*Scaleless specimen*	4500 USD
Bitis armata	750–800 EUR
Bitis caudalis	250–500 EUR
*Namibia*	350 EUR
*Rosh Pinah*	200–700 EUR
Bitis cornuta	200–800 EUR
*Springbok*	800 EUR
*Lüderitz*	1000 EUR
Bitis gabonica	200 EUR
Bitis rhinoceros	125–200 EUR
*Albino morph*	15,000–25,000 USD
Bitis nasicornis	400 EUR
Bitis parviocula	1200 EUR
Bitis peringueyi	250–1800 EUR
Bitis rubida	300–400 EUR
Bitis worthingtoni	300–700 EUR
Bitis xeropaga	500–700 EUR

**Fig. 4:**
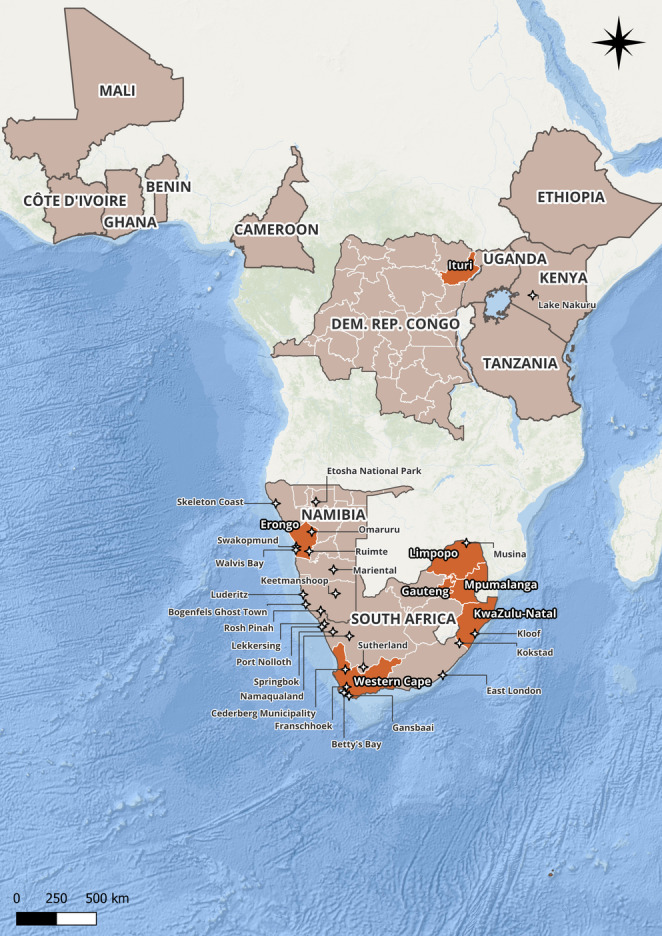
Named localities in the *Bitis* pet trade. Source: Created in QGIS by author with data from simplemaps.com

**Fig. 5:**
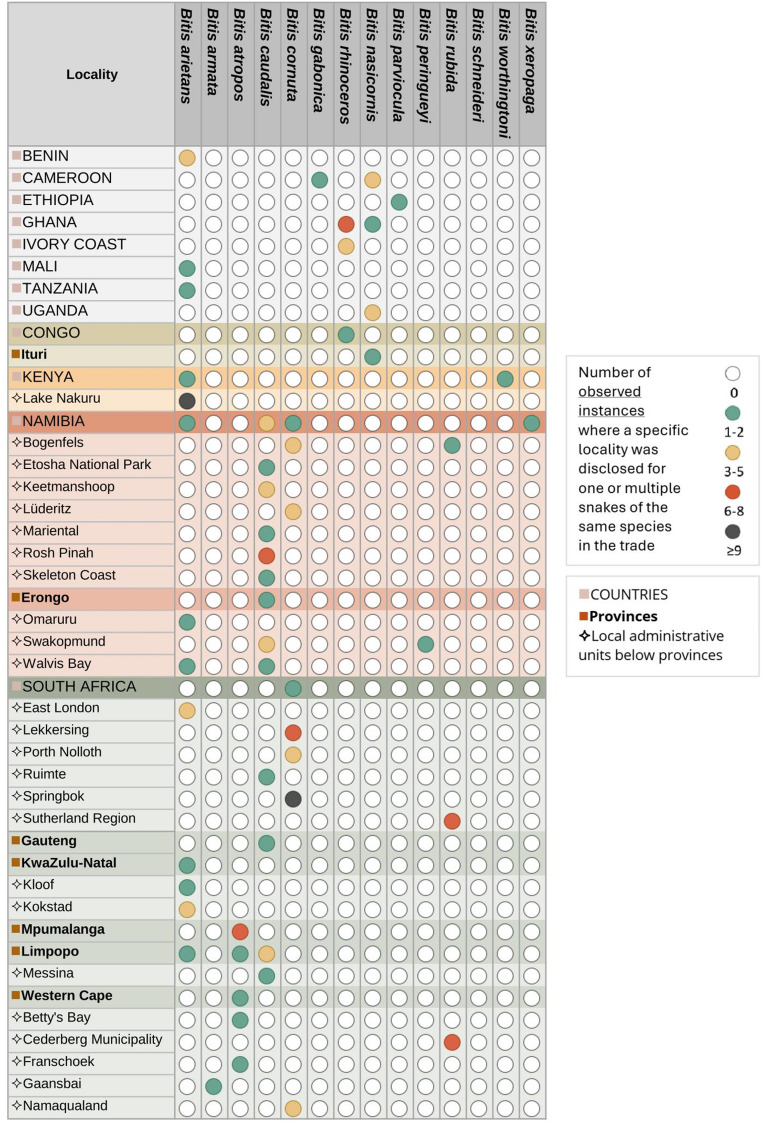
*Bitis* species and associated localities in the trade. Source: Created in Excel by author

Price depends on various factors beyond species, including locality (geographic origin of the species, which is frequently tied to specific phenotypes, see Figs. 1 and 2), mutations (such as albino or scaleless specimens) and market demand. A clear theme in my observations was that the monetary value of these factors is constantly negotiated in the market between practitioners in the trade. This can lead to inaccurate pricing estimates; with many noting that *Bitis* often sell for less than advertised. Prices can rise significantly in cases of specific phenotypes. Prices were often not displayed, and localities were sometimes labelled, sometimes not. Regardless, breeders familiar with *Bitis* could often identify these localities (see Figs. 4 and 5 for a comprehensive overview of *Bitis* species and localities in trade).

### Localities and behaviour

However, the value of *Bitis* goes beyond money, and *Bitis* with low prices can still be highly valued in less tangible ways. Locality-specific coding of my data showed that these geographic markers function as both aesthetic and identity-based signals within the community, shaping perceptions of legitimacy and value.

Some wildlife in the trade, such as crayfish, are referred to as “ornamental species” (Olden and Carvalho 2024, p. 1), rather than companion pets, due to their largely aesthetic appeal. Much of the value of *Bitis* appears to be related to their appearance, while reactions to and sometimes even interactions with humans are still vital aspects, making the term “ornamental pet” an apt descriptor. Ornamental factors include patterns, colours and slightly varying shapes and largely varying sizes, all of which vary by specific species as well as the geographic locality of that species. *Bitis*, like other animals, have different phenotypes, i.e. patterns and colours, depending on their location of origin on the African continent (see Fig. 1. and Fig. 2). Research on freshwater crayfish echoes similar implications, as their appeal, is linked to their phenotypes, among other factors (Chucholl and Wendler 2017, p. 199; Olden and Carvalho 2024, pp. 6–12). Besides species and gender, shared *Bitis* images or advertisements often, but not always, contain locality details, specifying either the country, the region, or specific towns of origin (see Figs. 4 and 5 for observed localities). Besides serving as an aesthetic differentiator among species, localities also provide buyers with insights into the environments in which the species may flourish. *Bitis* breeders have shown a preference for natural colour variations over artificially bred colours and strive to maintain the purity of specific localities rather than engage in crossbreeding. This may necessitate the sporadic introduction of wild-caught animals to avoid excessive inbreeding (Egan and Grant 1993). Similar to this preference for keeping localities pure, hybridisation between different *Bitis* species was considered interesting but not practised by breeders. Although crossbreeding occurs in the wild, breeders generally consider it less desirable because it can lead to infertile offspring. Notably, I found no localities disclosed for *Bitis schneideri*. Figure 4 depicts the localities present in trade and subject to human trading activities, providing a map of socio-ecological interactions involving *Bitis* as exotic pets, rather than just their geographical distribution.

Although venomous snakes are ornamental rather than companion animals, behaviour is another factor. While *Bitis* are generally considered calm due to being ambush hunters (Miller et al. 2015, pp. 2–3), all *Bitis*, from birth, have venom that is cytotoxic and cardiotoxic; depending on the amount of venom dispersed and the size of the snake, death and permanent disabilities can be the consequences of a *Bitis* bite (Dalhat et al. 2023, p. 4; Husain et al. 2023; Megale et al. 2020, pp. 1–10). Despite this danger and the fact that they are ornamental by most, some owners still engage in direct physical interactions with *Bitis*. Handling venomous snakes without the aid of hooks or gloves is a point of contention online and has its own hashtag (#freehandling). In interviews, many experienced keepers frowned on this practice but agreed that some very experienced and skilled handlers can do it safely if they are careful, though there is still a high risk of getting bitten one day. It was generally felt that social media posts showing free-handling of snakes were displays of expertise, bravery and, inadvertently, ego.

### Rarity, care, and market desirability

Rarity in relation to value is a complex and sometimes contradictory factor in the *Bitis* trade. In general, the relationship between rarity and price is a complex and evolving economic concept (De Bont 2024, pp. 36–40), and there are also concerns that the monetary value of wildlife can fuel exploitation and drive species below critical thresholds (Bulte 2003, pp. 34–35). In the wildlife trade, including the exotic pet market, this relationship, is often challenging to untangle but appears to be influenced by societal beliefs, individual values and interpretations of factors such as regulations and media portrayals (Harrington et al. 2019, pp. 33–35; Harris et al. 2017, p. 403; Hausmann et al. 2023, pp. 3–6). While some *Bitis* garner significant attention online due to their rarity, this does not necessarily translate into increased monetary value or market desirability. *Bitis* species that are rare in the wild often occupy particular environmental niches, and profitability is often hampered when species have complex care requirements. For reptiles in general, these needs often include costlier setups, including specific UV (ultraviolet) lights, humidifiers, and temperature controls for both ground and air. Rarity can also mean that less is known about the snake’s needs, increasing the risk of mortality and thereby deterring responsible keepers or those hoping to profit from breeding. In addition, the world of taxonomy and phylogeny is in a constant state of flux, evidenced by the frequent introduction or dissolution of taxa and fluctuating proposed relationships among various species and families, coupled with ongoing disputes among specialists in the field (Perry et al. 2020, p. 563; Segniagbeto et al. 2011, pp. 351–352). Similarly, not everyone in the *Bitis* community is convinced that species like *Bitis albanica* or *Bitis inornata* are rare, as they could be reclassified as subspecies of a more common *Bitis*.

### Trends and exceptions

According to interviewees, while there have been no persistent trends over time, subtle shifts, such as a decreased preference for large-bodied adders in the European trade, have been noted. However, *Bitis gabonica* (Fig. 3) and *Bitis rhinoceros*, as the largest *Bitis* examples, have retained their status as showpieces, even if they are less frequently bred. Due to pattern and size, *Bitis gabonica* is a staple among private keepers who maintain more diverse collections of large venomous snakes, which also frequently include cobras and rattlesnakes. An examination of online records (“Zootierliste 2024) also reveals a predominance of *Bitis arietans*, *Bitis gabonica* and *Bitis rhinoceros* within zoos compared to other *Bitis* species. This could be attributed to their size, which makes them more impressive for visitors, thereby increasing their value in this branch of the *Bitis* trade.

A notable outlier, however, is *Bitis parviocula*, which is scarce in the trade due to difficulties associated with breeding it. The first rigorous research on successful captive breeding in decades was recently published by London Zoo (Kane et al. 2022). *Bitis parviocula* was often mentioned by keepers as the *Bitis* they would most like to acquire, despite its having more complex needs than some other *Bitis*. Apart from its relative rarity, *Bitis parviocula* is distinctive for being the only *Bitis* species exhibiting a bright green and black pattern. Similarly, *Bitis heraldica* and *Bitis harenna* attract significant interest online due to their elusiveness in the wild. There have only been a handful of confirmed sightings (Gonçalves et al. 2019, p. 243; Gower et al. 2016, p. 54). However, neither *Bitis* heraldica nor *Bitis* harenna were present in the trade at the time of the study, according to my interviews and online observations.

The only significant market shift impacting the *Bitis* trade is the growing preference for artificially bred, unique, and often expensive colour and pattern variations of easy-to-breed species (Valdez 2021, p. 7). These variations, commonly called morphs or designer morphs, are popular among species such as ball pythons, leopard geckos, and corn snakes (Valdez 2021, p. 7). Along with this trend, investing in wildlife has become increasingly popular in recent years as a means of diversifying portfolios and offsetting potential losses in other financial markets (Di Minin et al. 2022, pp. 3–4; Zhu and Zhu 2024, p. 2). Breeding designer morphs has become an investment instrument for some, with certain ball pythons fetching prices around 30,000 USD and potentially more.

### Speculative value

While the *Bitis* community traditionally favoured natural patterns and colours, the successful captive breeding of *Bitis rhinoceros* albino morphs in South Africa may indicate a shift. These morphs have generated considerable interest in the market, with specimens fetching high prices, as noted by buyers on social media. This trend towards morphs is generally pronounced in the United States, a region known for its interest in unnatural colour variants and controversial breeding practices, such as scale-less snakes (Collis and Fenili 2011, pp. 15–17). Some buyers actively consider the investment potential of morphs; for example, they may view the initial investment of around 22,000–25,000 USD for an albino *Bitis rhinoceros* as a vehicle for return on investment through breeding and selling the subsequent offspring. However, such ventures carry significant risks, including breeding failures, non-viability of offspring, or premature deaths. Additional complexities stem from the wildlife market being driven by demand rather than supply (Hatten et al. 2024, p. 9; Zheng et al., 2024, pp. 2–3). Despite these challenges, high prices and the market trend towards morphs suggest the likelihood of future attempts to breed colour variations in other *Bitis* species, just as this was once considered impossible for *Bitis rhinoceros*, before the successful breeding attempts.

### Foreignness and symbolic associations

Physical artefacts such as postcards, stamps, collector’s cards, books, and dated advertisements featuring *Bitis* snakes indicate a relatively low monetary valuation of *Bitis*. The foreignness of *Bitis* appeared to be its primary appeal. In South Africa, keeping *Bitis* is even more of a niche interest than in Europe or the United States, with non-native reptiles attracting more attention overall. Interviewees confirmed that the general reptile-keeping community has a stronger fascination with foreign species than with native ones, even in places where it is legal to keep native species. Some interviewees stated that some people had even traded African adders for their European snakes in the past, showing that the appeal of foreignness works in both directions.

This theme of foreignness consistently appeared in the data on motivations for keeping *Bitis*, where the animals functioned as markers of place, memory, and distinction. Foreignness can also refer to the divide between life in suburbia, where humans reside and experiences in nature, where *Bitis* are encountered. Several interviewees, both in Europe and Africa, said snakes often serve as souvenirs of natural encounters during their travels. These reptiles not only symbolise appreciation for the natural environments they inhabit but also serve as living reminders of the snakes they have encountered in the wild, providing a tangible connection to those experiences. Based on these encounters or on photos shared by other *Bitis* enthusiasts showing the snakes in their natural habitats, keepers often endeavour to replicate the ideal habitat for these snakes within their enclosures. This process enhances the snake’s welfare to some degree but also caters to the keepers’ aesthetic preferences.

These patterns of value-making inform not only market outcomes but also the lived moral and practical logics that underpin the governance challenges that will be discussed later in this article.

## Socio-ecological learnings and governance in practice

Hull et al. (2023, pp. 4387–4395) show how work that links social dynamics with wildlife ecology is reshaping how scholars understand human–wildlife systems: he points to the need to push the current boundaries of this practice. My findings demonstrate the value of extending this approach to exotic pets. Socio-ecological practices research seeks to conceptualise the connections between human practices and nature, as well as the deliberate regulation of these interactions by policymakers (Sattlegger et al. 2025, pp. 6–8). In this sense, socio-ecological practice research focuses on what practitioners actually do in real-world human–environment systems, why they do it, and how these interventions unfold under ecological, social, and ethical constraints (Xiang 2019, p. 7). Drawing on the mechanics and value constructions previously discussed, I position the *Bitis* trade as an active participant within a dynamic socio-ecological system that interacts with regulations, rather than as a passive subject of them. Because robust population data for most *Bitis* species are currently limited, I do not offer prescriptive policy recommendations; instead, I outline governance implications that follow directly from observed trade practices and value-making dynamics and reflect on what this case contributes to socio-ecological practice research more broadly. For socio-ecological practice research, this illustrates how regulatory effects emerge not simply from legal texts, but from the behaviours based on the values held by humans in the trade as well as the restraints and possibilities of the context within which they operate. What appears as regulatory “failure” at the policy level may, from a practice perspective, reflect misalignments between governance instruments and the routines through which trade is conducted by its participants.

### National governance fragmentation and Socio-ecological effects

The trade practices and value systems in the *Bitis* trade described in the previous sections operate in a complicated and sometimes contradictory legal landscape, from the local to the international (Boniface 2016, pp. 146–148; Gabehart et al. 2024, pp. 20–28; Gebhardt-Brinkhaus 2023; Haenlein and Smith 2017, pp. 3–6; Legal Atlas, 2024; Mitsilegas et al. 2022, p. 31). In Europe, federal systems such as Germany’s have produced patchworks of venomous-animal regulations and uneven enforcement across states (Gabehart et al. 2024, pp. 20–28; Gebhardt-Brinkhaus 2023). In South Africa, national frameworks such as NEMBA and TOPS intersect with divergent provincial permitting regimes, creating the aforementioned opportunities for mislabelling and “province hopping” (Hübschle 2017, pp. 188–191), as *Bitis* collected in one province can be moved to another, where export permits can be obtained, and species are harder to verify.

From a socio-ecological perspective, these are not simply technical compliance issues. These laws have shaped who can and wants to participate in trade, on what terms, and with what incentives. European and North American breeders, who can command higher prices than local South African keepers, have incentives to navigate complex export regulations and are able to exploit loopholes. At the same time, some interviewees felt that restrictive South African regulations may limit opportunities for local breeders and venom laboratories to develop and maintain expertise in indigenous *Bitis*, arguably reproducing the kinds of unequal burdens and benefits that political ecologists have long documented in conservation regimes elsewhere (Adams and Hutton 2007, pp. 156–160; Hübschle and Margulies 2024, pp. 4–5; Massé et al. 2017, pp. 20–25). In their study of Himalayan wildlife “problems”, Khatri et al. (2024, p. 5) further illustrate how conservation policies can unintentionally redistribute costs and benefits in ways that actually erode local collective action and generate new conflicts; similar dynamics may play out in the *Bitis* trade if regulatory complexity alienates those actors who currently sustain legal, transparent trade channels and comply with regulations.

### CITES listings and the limits of rule-based governance

CITES listings are the most common framework for regulating the international wildlife trade. A potential intervention for South Africa would therefore be to list selected *Bitis* species in CITES. Only the Kenyan *Bitis worthingtoni* is listed in CITES Appendix II, requiring international export permits (“Wildlife Trade on the Internet | CITES,” 2023), though at the 20th Meeting of the Conference of the Parties, the decision was made to prohibit the trade in the Ethiopian *Bitis harenna* and *Bitis parviocula* by adding them to CITES Appendix I (*Summary record of the eleventh session for Committee I* 2025, p. 4), based on research showing the continued circulation of *Bitis parviocula* on the market and the potential for *Bitis harenna* to enter that market (Heim 2025, pp. 188–192; IUCN 2019, p. 151). How breeders will react to the presence of this desirable and less common *Bitis* in the pet trade remains to be seen. Any attempts to maintain the current captive population will test how successful captive breeding of this species has become.

From a socio-ecological practice perspective, this highlights the limits of interventions that target formal rules without engaging the value-making practices through which demand, legitimacy, and desirability are produced. In the *Bitis* case, some actors already display considerable skill in navigating, or even circumventing, African, European, and US regulations. I found nothing to indicate that *Bitis* are traded just because it is possible due to lax regulation; a trade in *Bitis* has emerged because they are valued as pets. The risk that tighter international restrictions might displace activity into less visible channels is worth taking seriously. Other research, such as that examining the dried seahorse trade, illustrates how persistent demand and complex supply chains can sustain illegal or unsustainable trades, despite identification tools and monitoring efforts (Vaidyanathan et al. 2024, pp. 7–11). In this context, blunt increases in regulatory strictness risk triggering what Biggs et al. (2013, p. 1038) term a “supply-and-demand extinction vortex”: if legal captive breeding becomes too onerous or unprofitable, experienced breeders may scale back or exit the market, shrinking legal supply and potentially increasing incentives for covert wild collection. When considering the existing trade structures, such suspensions can, in turn, stimulate laundering and black-market activity, as has been documented for other wildlife commodities such as seahorses (Vaidyanathan et al. 2024, pp. 7–11) and in broader debates on trade bans (‘T Sas-Rolfes et al. 2019, pp. 208–209; Mialon et al. 2022, pp. 2–12; Weber et al. 2015, pp. 390 − 191). Broad and indiscriminate trade restrictions, including comprehensive export or import bans, can inadvertently alter trading behaviours in detrimental ways. Such measures may intensify species threats by signalling anticipated scarcity within markets, thereby driving prices upward and enhancing incentives for illegal exploitation (Hiller & ’t Sas-Rolfes, 2025, p. 16). However, in the case of niche exotic pets such as *Bitis*, the ban on *Bitis parviocula* trade will be a test of how highly trade participants value this species, which, while desirable for its appearance, is also considered difficult to care for and breed. Treating trade regulations as tests of demand neglects that changes in practices arise not solely from governance, but from the interplay of developments across the broader network of practices and arrangements (Sattlegger et al. 2025, p. 6).

### Genetic monitoring as a Socio-ecological intervention

Given the small size of the *Bitis* market and the presence of only a few successful breeders, one governance pathway is to combine limited harvesting quotas with genetic tools that distinguish captive-bred from wild-caught animals. Under conditions of low demand, tightly controlled quotas could be paired with a DNA database to reduce laundering risks and signal new shared norms around responsible keeping. Hogg et al. (2018, pp. 238–243) show that genetic markers can be used to differentiate known captive-bred individuals from privately held specimens, and that permit requests declined after DNA sampling became mandatory. Campbell et al. (2019, pp. 3–10) demonstrate that some exotic pet markets are indeed anchored in small founder populations, suggesting that verifiable captive breeding can underpin legal trade. In the *Bitis* case, the trade is sufficiently small that a database is technically feasible, and several interviewees expressed support for testing.

Approached from a socio-ecological standpoint, genetic monitoring does more than police supply chains: it alters relations among actors, values, and infrastructures. A DNA-based system would formalise particular breeder networks as governance partners, embedding them more deeply in regulatory structures while potentially excluding smaller keepers or those without access to sampling infrastructure. According to Naito et al.’s (2022, p. 176) integrative framework for transformative social change, this is a structural intervention (creating new materials, competences, and institutional rules) that could also encourage private actions (breeders voluntarily submitting samples) and social-signalling actions (public endorsement of “clean” captive lines). The result would not simply be improved enforcement but a shift in relational values: what counts as responsible practice, desirable breeding outcomes, and acceptable interactions with wild stock. This solution would require political will and resources, though it would not require source countries to sponsor non-detriment findings (NDFs) or other biological and legal assessments (Robinson and Roberts 2024, p. 2; Wyatt 2021, pp. 82–95). Private breeders might take the opportunity to use genetic verification to stand out from other breeders and verify their status as legitimate breeders, even on a voluntary basis.

Yet, as Rizzolo (2021, pp. 11–12) and (Tensen 2016, pp. 289–290) warn, expanding captive breeding can actually stimulate demand by making ownership acceptable to those who were hesitant before. In the *Bitis* market, designer morphs – such as albino *Bitis rhinoceros* – materialise a speculative aesthetic that increases desire and normalises commodification observed in the overall reptile market (Valdez 2021, pp. 9–11). In Naito et al.’s (2022, p. 183) terms, this is the value change pathway operating negatively: structural innovations produce new meanings and motivations that encourage consumption beyond what captive breeders can supply. The same procedures intended to close laundering pathways can therefore generate fresh socio-ecological risks by increasing market visibility, status signalling, and speculative investment.

### Implications for Socio-ecological practice research

This study shows how analysing the mechanics and value perceptions of a niche wildlife trade can advance socio-ecological practice research. In the *Bitis* trade, value-making emerges as a central socio-ecological practice rather than a background social factor. Values are enacted through concrete practices such as selective breeding, sourcing decisions, and the online circulation of animals and expertise. These practices directly shape how *Bitis* are collected, bred, and traded, and therefore how ecological pressures accumulate over time. Treating value-making as practice makes visible how ecological consequences arise through routine decisions that actors do not necessarily frame as conservation-relevant.

My findings also underline the importance of approaching governance as something enacted through everyday coordination rather than through formal compliance alone. Permitting systems, provincial and national regulations, and international listings all matter, but their effects depend on how traders interpret rules, navigate regulatory boundaries, and mobilise expertise to classify animals as legal or captive-bred. Practices such as province hopping and mislabelling illustrate how working around governance is folded into existing routines. From a practice-based perspective, what appears as regulatory failure at the policy level often reflects misalignments between governance instruments and the practices through which trade is organised.

By focusing on a small, specialist market, this study further demonstrates that socio-ecological complexity is not limited to large-scale or high-value wildlife trades. Despite involving relatively few actors and low volumes, the *Bitis* trade displays intricate interactions between legality, expertise, valuation, and ecological risk. This suggests that niche systems are analytically productive sites for socio-ecological practice research, as their practices and value dynamics are more visible and traceable than in larger, more opaque markets.

## Conclusion

In this article, I examined the *Bitis* viper pet trade as a socio-ecological system in which trade practices, value-making, and governance arrangements are closely intertwined. Using a zooming-in, zooming-out approach, I traced how *Bitis* move through African–European trade networks via everyday practices that shape the conditions under which *Bitis* are collected, kept, bred, and traded, and how these practices interact with regulatory frameworks in ways that are often uneven, ambiguous, and variably interpreted on the ground. Rather than treating traders and keepers solely as external pressures on conservation, the findings illustrate how they act as situated participants within the socio-ecological system.

Empirically, this study contributes a detailed account of a small-scale, internationally connected wildlife trade that has rarely been examined in depth. Conceptually, it demonstrates the usefulness of a practice-based socio-ecological perspective for understanding wildlife trade governance. Instead of focusing only on policy design or biological impact, this approach makes visible how governance is enacted through everyday routines and infrastructures that shape ecological outcomes over time.

At the same time, the scope of the findings is constrained by available data. Robust population estimates and offtake data for most *Bitis* species remain limited, and this study does not claim to establish causal relationships between trade practices and population decline in the wild. For this reason, I do not offer prescriptive policy recommendations. Instead, I identify patterns and points of tension that are relevant to understanding how current governance arrangements interact with trade practices and value constructions. Future research would benefit from combining practice-based approaches with targeted ecological monitoring, improved permit and seizure data, and comparative work across other niche reptile trades.

*The funding for this research was provided by the European Research Council (ERC) under the European Union’s Horizon 2020 research and innovation programme (grant agreement n° 804851).*

## Data Availability

No datasets were generated or analysed during the current study.

## Appendix

Semi-Structured Interview Checklist:

Topic 1: Pathways into Bitis Breeding/Keeper Practice

How did you start breeding or keeping Bitis?

Who do you rely on (mentors, networks, suppliers) to support your work?

What tools, equipment, or techniques are essential to your practice?

Topic 2: Motivations and Operational Decisions

Why did you choose to specialise in Bitis?

How quickly can you adjust breeding or sales strategies in response to market trends?

Do you use (or need) wild-caught animals, and why?

Are there concerns related to inbreeding?

Are there aspects of the Bitis community or market you find challenging or problematic?

Topic 3: Social and Market Dynamics of the Bitis Community

How would you describe the community surrounding Bitis?

How does it compare to other reptile-breeding communities?

Is the community stable and established, or do newcomers frequently enter and exit?

How does community structure influence what you breed, trade, or sell?

Are agreements typically formal (contracts) or informal (verbal trust-based)?

To what extent is the community international?

## Footnotes

1 CITES is an international agreement regulating and/or prohibiting the trade in CITES-listed species.
